# Diagnostic accuracy cohort study and clinical value of the Histoplasma urine antigen (ALPHA Histoplasma EIA) for disseminated histoplasmosis among HIV infected patients: A multicenter study

**DOI:** 10.1371/journal.pntd.0006872

**Published:** 2018-11-05

**Authors:** Pedro Torres-González, María Dolores Niembro-Ortega, Areli Martínez-Gamboa, Víctor Hugo Ahumada-Topete, Jaime Andrade-Villanueva, Javier Araujo-Meléndez, Alberto Chaparro-Sánchez, Brenda Crabtree-Ramírez, Sofia Cruz-Martínez, Armando Gamboa-Domínguez, Oscar I. Flores-Barrientos, Jesús Enrique Gaytán-Martínez, Luz Alicia González-Hernández, Christian Hernández-León, Víctor Hugo Lozano-Fernandez, Marisol Manríquez-Reyes, Martin Magaña-Aquino, Pedro Martínez-Ayala, Juan Pablo Ramírez-Hinojosa, Andrea Rangel-Cordero, Norma Erendira Rivera-Martínez, Edgardo Reyes-Gutiérrez, Gustavo Reyes-Terán, Patricia Rodríguez-Zulueta, Jesús Ruíz-Quiñones, Janeth Santiago-Cruz, Nancy Guadalupe Velázquez-Zavala, José Sifuentes-Osornio, Alfredo Ponce de León

**Affiliations:** 1 Department of Infectious Diseases, Instituto Nacional de Ciencias Médicas y Nutrición Salvador Zubirán, Tlalpan, Mexico City, Mexico; 2 Centro de Investigación en Enfermedades Infecciosas, Instituto Nacional de Enfermedades Respiratorias Ismael Cosío Villegas, Tlalpan, Mexico City, Mexico; 3 HIV Unit, Hospital Civil de Guadalajara “Fray Antonio Alcalde”, Guadalajara, Jalisco, Mexico; 4 Department of Internal Medicine, Hospital Central Dr. Ignacio Morones Prieto, San Luis Potosí, San Luis Potosí, Mexico; 5 Adult Infectious Diseases Department, Hospital de Infectología del Centro Médico Nacional “La Raza”, Instituto Mexicano del Seguro Social, Atzcapotzalco, Mexico City, Mexico; 6 Department of Infectious Diseases, Hospital Regional de Alta Especialidad de Oaxaca, HRAEO, San Bartolo Coyotepec, Oaxaca, Mexico; 7 Department of Pathology, Instituto Nacional de Ciencias Médicas y Nutrición Salvador Zubiran, Tlalpan, Mexico City, Mexico; 8 Intensive Care Unit, Department of Internal Medicine, Hospital “Dr. Juan Graham Casasus”, Villahermosa, Tabasco, Mexico; 9 Area of Infectious Diseases, Department of Internal Medicine, Hospital General de Puebla “Dr. Eduardo Vazquez Navarro”, Puebla, Puebla, Mexico; 10 Department of Internal Medicine, Hospital de Alta Especialidad de Veracruz, Veracruz, Veracruz, Mexico; 11 Infectious Diseases Department, Hospital General Dr. Manuel Gea González, Tlalpan, Mexico City, Mexico; 12 Department of Medicine, Instituto Nacional de Ciencias Médicas y Nutrición Salvador Zubirán, Tlalpan, Mexico City, Mexico; Universidad de Antioquia, COLOMBIA

## Abstract

**Background:**

The Histoplasma urine antigen (HUAg) is the preferred method to diagnose progressive disseminated histoplasmosis (PDH) in HIV patients. In 2007, IMMY ALPHA Histoplasma EIA was approved for clinical for on-site use, and therefore useful for regions outside the United States. However, ALPHA-HUAg is considered inferior to the MVista-HUAg which is only available on referral. We aim to evaluate the diagnostic accuracy of ALPHA-HUAg.

**Methodology/Principal findings:**

We conducted a multicenter, prospective, diagnostic test study in two secondary and eight tertiary-care facilities in Mexico. We included HIV patient with PDH suspicion and evaluated ALPHA-HUAg diagnostic accuracy using as reference standard the *Histoplasma capsulatum* growth on blood, bone marrow, and tissue cultures or compatible histopathologic exam (PDH–proven). We evaluated the results of 288 patients, 29.5% (85/288; 95% confidence interval [CI], 24.3–35.1) had PDH. The sensitivity of ALPHA-HUAg was 67.1% (95% CI, 56–76.8%) and the specificity was 97.5% (95% CI, 94.3%-99.1%). The positive likelihood ratio was 27.2 (95% CI; 11.6–74.4). In 10.5% of the PDH–proven patients, a co-existing opportunistic infection was diagnosed, mostly disseminated *Mycobacterium avium* complex infection.

**Conclusions/Significance:**

We observed a high specificity but low sensitivity of IMMY-HUAg. The test may be useful to start early antifungals, but a culture-based approach is necessary since co-infections are frequent and a negative IMMY-HUAg result does not rule out PDH.

## Author summary

Histoplasmosis is an endemic mycosis in many regions of Latin America. In the HIV population it manifests as progressive disseminated histoplasmosis (PDH), an entity hard to diagnose since the causative fungi—*Histoplasma capsulatum*—is slow growing and requires advance biosafety for its handling. As an alternative, the diagnosis can be made by detecting the histoplasma urinary antigen, but only one kit is commercially available outside the US. We evaluated this kit (IMMY ALPHA Histoplasma EIA) among HIV patients with suspected and found a high specificity but low sensitivity (i.e., a positive histoplasma urine antigen is almost unequivocal, but a negative result does not rule out the PDH). However, we believe the test is useful since the positive likelihood rates show that a patient with PDH is 27 times more likely to have an IMMY-HUAg positive result; this may allow early targeted treatment.

## Introduction

Histoplasmosis is a mycosis endemic to many parts of Latin America, including Mexico. [1] The clinical presentation varies depending on the host´s immune status; for HIV-infected patients in advanced stages of the disease, it usually presents as progressive disseminated histoplasmosis (PDH), and it is fatal unless timely diagnosed and treated.[2] The standard of reference for PDH diagnosis is the isolation of *Histoplasma capsulatum* from a non-respiratory sample—typically bone marrow or peripheral blood—or the visualization of yeast-like structures in the histopathologic examination.[3] Unfortunately, *H*. *capsulatum* is a slow-growing fungus, and cultures may require up to six weeks of incubation.[4] Moreover, the handling of *H*. *capsulatum* isolates requires advanced biosafety facilities which are scarce in Latin America.[5] Because of this, and the absence of reliable and simple methods to establish the diagnosis, some have advocated that histoplasmosis is underreported in Latin America and since, a neglected disease. [6] In the United States, histoplasmosis is also endemic, and its PDH presentation is commonly diagnosed by the detection of Histoplasma urine antigen (HUAg). This test, introduced in the late 80´s was performed, until recently, by a single central laboratory (MiraVista Diagnostics).[7] In 2007, a new HUAg detection kit was released approved by the Food and Drug Administration (FDA) for clinical use and commercialized for on-site use (IMMY ALPHA Histoplasma EIA Test Kit, IMMY, Inc.). [8] Nevertheless, the diagnostic accuracy of ALPHA-HUAg is considered inferior to the centralized test (MiraVista Urine Antigen test).[9] However, multicenter comparisons of these tests against fungal cultures and histopathology are lacking. For regions outside the United States, an on-site HUAg test is preferred for logistics, costs and turn-around time of results. Therefore, we decided to prospectively and independently evaluate the diagnostic accuracy of ALPHA-HUAg for the PDH diagnosis among HIV-infected patients.

## Methods

### Patients and settings

We conducted a multicenter, prospective, diagnostic test study in two secondary and eight tertiary-care facilities from seven states of Mexico. From December 2015 to October 2017, patients older than 18 years with previous HIV diagnosis—or confirmation of diagnosis during the hospital stay—and suspicion of PDH were identified by the study´s physicians—all of them Infectious Diseases specialists experienced in the management of HIV-infected patients—from each center, and hospitalized for study. Suspected PDH was defined as the presence of at least three of the following clinical signs and symptoms: fever, unintentional weight loss (>5% usual body weight over 6–12 months), diarrhea, peripheral lymphadenopathy, hepatomegaly, splenomegaly and skin lesions, mucosal lesions and at least one laboratory abnormality. We defined laboratory abnormalities as the presence of elevated aspartate aminotransferase (AST) (2-fold the upper limit), lactic dehydrogenase (2-fold the upper limit), elevated ferritin, bicytopenia or pancytopenia. For inclusion, at least a blood or a bone marrow culture in media supporting *H*. *capsulatum* growth (Bactec Myco F/ Lytic) and 20 ml of urine were required; according to the on-site physician´s criteria, additional tissue samples from diverse anatomical sites were obtained for culture and histopathology. Demographic and clinical data—history of other opportunistic infections (OI), HIV diagnosis date, antiretroviral drug treatment, CD4+ lymphocytes count and plasma HIV RNA—, were collected in a case report form and sent along the biological samples to the study´s central laboratory in Mexico City. Preliminary and definitive results were informed to the on-site physician as soon as possible. We defined proven PDH cases as the growth *H*. *capsulatum* or the observation of yeast cells intracellularly or extracellular and massive macrophage infiltration and scattered lymphocytes by Grocott´s methamine and Periodic acid-Schiff stains during the histopathology exam, in any sample besides the lungs. In accordance with the European Organization for Research and Treatment of Cancer/Invasive Fungal Infections Cooperative Group and the National Institute of Allergy and Infectious Diseases Mycoses Study Group definitions, [10] we classified as PDH–negative cases as the absence of *H*. *capsulatum* growth in cultures or the absence of yeast-like structures in the histopathology exam regardless of whether other OI or AIDS-defining malignancies were diagnosed. Since we are testing HUAg, cases with a positive test in absence proven PDH were considered as false positives—instead of possible PDH—and cases with a negative result of HUAg in the presence of proven PDH, as false negatives. Simultaneous diagnosis of HIV and PDH was considered whenever both infections were diagnosed within the same month.

### Microbiologic methods

Every center was provided with the materials for samples collection, and these were shipped to the study´s central laboratory for processing. At least 5 mL of peripheral blood were inoculated in a BD BACTEC Myco F/ Lytic (Becton Dickinson, and Company, Franklin Lakes New Jersey, USA) vial; for bone marrow aspirates, a similar amount (5 mL) was inoculated in a Bactec Myco F/ Lytic and a Bactec Peds Plus F (Becton Dickinson, and Company) (3 ml), and 1 ml in a Löwenstein-Jensen and Sabouraud solid media. Biopsies for culture were transported in Stuart media and for histopathological analysis, in 10% formaldehyde. Bactec Peds Plus F and Bactec Myco F/Lytic vials were incubated in the semi-automated FX apparatus (Becton Dickinson, and Company, Franklin Lakes New Jersey, USA) for seven and 42 days, respectively. If determined positive by the apparatus, vials were retrieved for Ziehl-Neelsen and Gram staining and sub-cultured in MGIT liquid media (Becton Dickinson, and Company), Sabouraud solid media and blood sheep agar. Biopsies for culture were macerated, suspended in saline solution 0.9% and distributed in four vials (Bactec Aerobic/F, Bactec Anaerobic/F, Bactec Peds Plus/F and Bactec Myco F/Lytic) and incubated for seven, 21, 30 and 42 days respectively. All isolates were fully characterized by conventional methods and in mycobacterial species using DNA probes (HAIN Lifescience GmbH, Nehren, GER). The *H*. *capsulatum* isolates were identified by colony morphology at 25–30°C: white to brown or pinkish with fine, dense cottony texture and microscopically: the presence of large thick-walled, round macroconidia (7 to 15 m in diameter) with tuberculate, knobby or short cylindrical projections. The central laboratory of this study is accredited by the College of American Pathology for bacterial, fungal and mycobacterial identification. Urine samples—collected upon inclusion to the study by any method and at time of the day—were transported at 4 C° and upon arrival to the central laboratory, frozen at -20°C and processed for HUAg in batches every two weeks. The HUAg was performed using the IMMY ALPHA Histoplasma EIA test kit (IMMY, Inc. Norman OK)—currently, the only Food Drugs Administration approved kit for on-site use—following the manufacturer´s instructions. These kits were purchased from the manufacturer. Laboratory technicians responsible for the HUAg reading and culture processing were blinded to the result of the rest of the samples. The pathologists in charge of the histopathologic examination were also blinded to the microbiology results and the HUAg. Incident HIV cases were diagnosed using the kit ARCHITECT HIV Ag/Ab Combo (Abbot, Wiesbaden, Germany).

### Statistical analysis

We described and compared the patient’s characteristics and clinical variables between PDH–proven patients, PDH–negative patients and the characteristics of the patients with HUAg false negative results. We used median and interquartile range (IQR) for continuous variables and frequencies and percentages for categorical variables and Mann-Whitney U and Pearson’s X^2^ or Fisher´s exact test as statistical tests accordingly. A *p*-value ≤ 0.05 was determined as significant. We calculated sensitivity, specificity, positive predictive value, negative predictive value and positive likelihood ratio and their 95% confidence intervals (95% CI) of HUAg using as a standard of reference the cases classified as PDH–proven. We used the STATA 11.0 software (StataCorp LLC, College Station, TX) to perform the statistical analysis.

### Ethical considerations

This protocol was reviewed and approved by the Institutional Review Board of the coordinator center (Instituto Nacional de Ciencias Médicas y Nutrición Salvador Zubirán, Comisión Institucional para la Investigación Biomédica en Humanos REF: 1626) and by the institutional review boards in each center. The study was conducted according to the principles expressed in the Declaration of Helsinki. Physicians collaborating in the study invited patients with suspected PDH to participate and, after explaining the aims of the study, if agreed, patients (or their relatives in the case of mental impairment or critical illness) signed an informed consent form.

## Results

**Fig 1:**
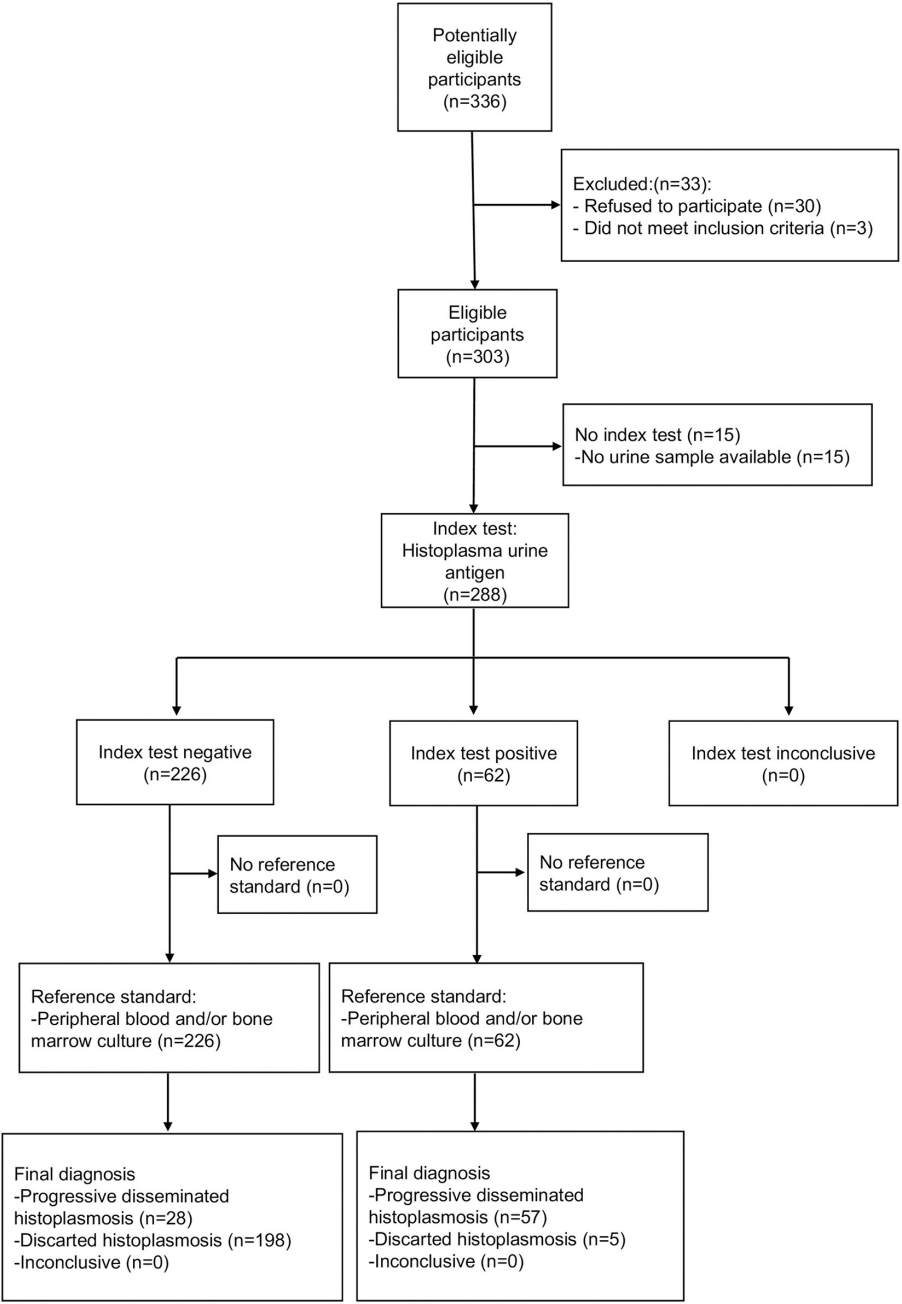
Study flowchart.

During the study period (23 months), 336 potentially eligible patients were assessed in the participant centers, 30 refused to participate in the study and three did not fulfill the inclusion criteria. The remainder 303 patients with suspected PDH signed the informed consent, but 15 were excluded because the urine sample was not available. We finally included 288 cases for this analysis. (Fig 1) From these patients, the central laboratory processed 1,068 samples: 277 blood cultures, 252 bone marrow aspirate cultures, 104 biopsies for tissue culture, 147 biopsies for the histopathologic exam, and 288 urine samples for HUAg detection. (Table 1).

**Table 1:** Microbiological and histopathological positivity for *Histoplasma capsulatum* of the samples processed from 288 patients with progressive disseminated histoplasmosis suspicion.

Anatomical site	*H*. *capsulatum* positive cultures	Histopathology positive for histoplasmosis
	No./N (%)	No./N (%)
Blood	56/277 (20.2)	-
Bone marrow aspirate	64/252 (25.4)	-
Skin and soft tissues	5/23 (22.7)	11/30 (36.6)
Gastrointestinal tract	0/20 (0)	1/30 (3.3)
Bone/bone marrow biopsy	1/21 (4.7)	17/50 (34)
Lymph nodes	5/21 (23.8)	5/16 (31.2)
Liver or spleen	0/10 (0)	0/10 (0)
Lung	1/6 (16.6)	2/8 (25)
Central nervous system	0/3 (0)	0/3 (0)
Total	132/633(20.8)	36/147 (24.4)

*H*. *capsulatum* grew in 20.2% (56/277) of the blood cultures, and in 25.4% (64/252) of the bone marrow aspirate cultures. From 82 patients we acquired at least one tissue culture, and *H*. *capsulatum* grew in 13.4% (11/82) from at least one sample per patient. From 103 patients, we also processed at least one biopsy for the histopathologic exam, and in 33.9% (35/103) we observed changes consistent with histoplasmosis from at least one sample per patient.

We classified 85/288 (29.5%) patients as PDH–proven, 28.2% (24/85) based on positive histopathologic exam and positive culture, 58.8% (50/85) by positive culture only and 12.9% (11/85) by positive histopathologic exam only.

### Clinical characteristics of proven and negative progressive disseminated histoplasmosis patients

The PDH–proven patients, were significantly younger and more frequently received more than 72 hrs of antifungal treatment before obtaining samples for the study in comparison with PDH-negative patients. They also presented a significantly larger proportion of weight loss, skin lesions, anemia, leukopenia, and thrombocytopenia while PDH-negative patients presented more frequently with peripheral lymphadenopathy. The median levels of total bilirubin, aspartate transaminase, lactic dehydrogenase, and ferritin were significantly higher among patients with proven PDH. Ten percent (9/85) of the proven PDH patients, were diagnosed with at least an additional OI or AIDS-defining malignancy, more frequently disseminated *Mycobacterium avium* complex (MAC) infection and Kaposi Sarcoma. Among the PDH-negative patients, we identified the presence of OI or AIDS-defining malignancy in 28% (57/203) of the cases, more frequently disseminated MAC infection and *M*. *tuberculosis* complex. (Table 2).

**Table 2:** Clinical characteristics of 85 proven progressive disseminated histoplasmosis patients among 288 suspected cases.

Characteristic	PDH–proven, No./N (%)	PDH–negative, No./N (%)	*p*-value
	n = 85	n = 203	
Demographics and HIV evolution			
Age; median (IQR)	33 (27–38)	36 (29–44)	0.031^b^
Gender (male)	68/85 (80)	176/203 (86.7)	0.149
Simultaneous HIV diagnosis	40/85 (47)	100/203 (49.2)	0.733
Months since HIV diagnosis^a^, median (IQR)	25.5 (5.2–75.8)	47 (8–110)	0.125^b^
Previous OI and HIV-treatment			
Previous tuberculosis	8/85 (9.4)	31/203 (15.2)	0.185
Previous disseminated MAC	2/85 (2.3)	6/203 (2.9)	0.776
Previous disseminated histoplasmosis	4/85 (4.7)	5/203 (2.4)	0.318
CD4+ lymphocytes (cells/mm^3^); median (IQR)	30 (9–62)	41.5 (14–86)	0.084^b^
Plasma HIV RNA (copies/mm^3^); median (IQR)	141,607 (38,669–474,905)	238,834 (31,237–808,567)	0.327
ART naïve	50/85 (58.8)	110/203 (54.1)	0.470
2 NRTI +1 NNRTI	21/35 (60)	49/93 (52.6)	0.459
2 NRTI + 1IP/r	8/35 (22.8)	26/93 (27.9)	0.560
Other ART	6/35 (17.1)	18/93 (19.3)	0.775
Empirical antifungal ≥ 72 hrs. previous to samples	20/85 (23.5)	28/203 (13.7)	0.043^b^
Initial admission to critical care	4/83 (4.8)	7/196 (3.5)	0.624
Symptoms			
Fever	81/85 (95.2)	180/203 (88.6)	0.079
Weight loss	80/85 (94.1)	172/203 (84.7)	0.028
Diarrhea	47/85 (55.2)	90/203 (44.3)	0.089
Peripheral lymphadenopathy	41/85 (48.2)	130/203 (64)	0.013
Hepatomegaly	55/85 (64.7)	123/203 (60.5)	0.512
Splenomegaly	40/85 (47)	93/203 (45.8)	0.847
Mucosal lesion	10/85 (11.7)	35/203 (17.2)	0.243
Skin or soft tissue lesion	28/85 (32.9)	40/203 (19.7)	0.016
Laboratory abnormalities			
Anemia	61/85 (71.7)	119/203 (58.6)	0.036
Leukopenia	50/85 (58.8)	87/203 (42.8)	0.013
Thrombocytopenia	46/85 (54.1)	73/203 (35.9)	0.004
Total bilirubin (mg/dL); median (IQR)	0.7 (0.5–1.5)	0.6 (0.4–1.1)	0.033^b^
Alanine transaminase (UI/L); median (IQR)	42 (26–70)	37 (20–58)	0.084^b^
Aspartate transaminase (UI/L); median (IQR)	102 (57–258)	47 (28–98)	<0.001^b^
Lactic dehydrogenase (UI/L); median (IQR)	985.5 (562.5–2,042.5)	300.5 (193–551)	<0.001^b^
Ferritin (ng/mL), media (IQR)	1,948.5 (1,367.8–10,203.8)	788.1 (432–2084.3)	0.045^b^
Radiographic Pulmonary Involvement	63/85 (74.1)	141/203 (69.4)	0.427
Other opportunistic infection/neoplasia diagnosed	9/85 (10.5)	57/203 (28)	<0.001
Disseminated MAC	4/85 (4.7)	24/203 (11.8)	–
Disseminated tuberculosis	0/75 (0)	20/203 (9.8)	–
Disseminated *Cryptococcus neoformans*	1/85 (1.1)	3/203 (1.4)	–
Kaposi Sarcoma	2/85 (2.3)	9/203 (4.4)	–
HIV lymphadenopathy	0/85 (0)	2/203 (0.9)	–
Lymphoma	1/85 (1.1)	6/203 (2.9)	–
Disseminated Citomegalovirus	1/85 (1.1)	4/203 (1.9)	–
Extraintestinal *Salmonella* spp infection	1/85 (1.1)	2/203 (0.9)	–

Abbreviation: PDH, progressive disseminated histoplasmosis; IQR, Interquartile Range; MAC, *Mycobacterium avium* complex; ART, Antiretroviral therapy; NRTI, Nucleoside Reverse Transcriptase Inhibitor; NNRTI, Non-Nucleoside Reverse Transcriptase Inhibitor; PI/r, Protease Inhibitor/ritonavir. If not otherwise indicated X^2^ test was applied.

^a)^ Excluding simultaneous HIV/disseminated histoplasmosis diagnosis;

^b)^ Mann-Whitney U-test.

### Sensitivity and specificity of Histoplasma urine antigen for the diagnosis of progressive disseminated histoplasmosis

Twenty-one percent of the patients (62/288) had a positive HUAg. The sensitivity of HUAg for the diagnosis of proven PDH was 67.1% (95% CI, 56%-76.8%) and the specificity 97.5% (95% CI, 94.3%-99.1%). The positive likelihood ratio was 27.2 (95% CI; 11.6–74.4), and the negative likelihood ratio was 0.33 (95% CI; 0.29–0.41). The sensitivity of HUAg for the diagnosis of culture-proven PDH was 73.9% (95% CI, 62.3%–83.5%) and the specificity 96% (95% CI, 92.8%–98.3%). The positive likelihood ratio was 19.8 (95% CI; 10.3–41.5) and the negative likelihood ratio of 0.27 (0.21–0.36). (Table 3). The sensitivity was 69.1% (95% CI; 64.1–69.1) for blood cultures; 82.1% (95% CI; 77.1–82.1) for bone marrow cultures and 72.9% (95% CI; 65.3–72.9) for the histopathological exam, in comparison with our standard of reference.

**Table 3:** Diagnostic accuracy of Histoplasma urine antigen among proven and culture-proven cases of progressive disseminated histoplasmosis.

	Proven PDH			Culture-proven PDH		
Histoplasma Urine Antigen	Positive, No. (%)	Negative, No. (%)	Total, No. (%)	Positive, No. (%)	Negative, No. (%)	Total No. (%)
Positive	57 (19.8)	5 (1.7)	62 (21.5)	54 (18.8)	8 (2.8)	62 (21.5)
Negative	28 (9.7)	198 (68.8)	226 (78.5)	19 (6.6)	207 (71.9)	226 (78.5)
Total	85 (29.5)	203 (70.5)	288 (100)	73 (25.3)	215 (74.7)	288 (100)
Sensitivity % (95% CI)	67.1 (56–76.8)			73.9 (62.3–83.5)		
Specificity % (95% CI)	97.5 (94.3–99.1)			96 (92.8–98.3)		
Positive predictive value % (95% CI)	91.9 (82.1–97.3)			87.1 (76.1–94.2)		
Negative predictive value % (95% CI)	87.6 (82.5–91.6)			91.6 (87.1–94.8)		
Positive likelihood ratio (95% CI)	27.2 (11.6–74.4)			19.8 (10.3–41.5)		
Negative likelihood ratio (95% CI)	0.33 (0.29–0.41)			0.27 (0.21–0.36)		

Abbreviation: PDH, progressive disseminated histoplasmosis, CI, confidence interval

### Clinical characteristics of proven progressive disseminated histoplasmosis cases with a Histoplasma urine antigen false negative result

Thirty-three percent (28/85) of the patients with proven PDH presented a false negative HUAg result. Among these, 21.4% (6/28) were diagnosed by culture and histopathology, 46.4% (13/28) by culture only, and 32.1% (9/28) by histopathology only. These patients were similar in age, the frequency of presenting symptoms, plasma HIV RNA, antiretroviral treatment, time since HIV diagnosis, and exposure to antifungal before obtaining the samples, but had a significantly higher median of CD4+ lymphocytes count and lower median of alanine transaminase, aspartate transaminase, and lactic dehydrogenase. Patients with a false negative result of HUAg also presented more frequently with an additional OI or AIDS-defining malignancy besides PDH. (Table 4).

**Table 4:** Comparison of clinical characteristics of proven progressive disseminated histoplasmosis with false negative results of Histoplasma urine antigen in HIV patients.

Characteristic	False negative, HUAg No./N (%)	True positive HUAg, No./N (%)	*p*-value
	n = 28	n = 57	
Demographics and HIV evolution			
Age; median (IQR)	31.5 (26–36.5)	34 (28–39)	0.199^b^
Gender (male)	24/28 (85.7)	44/57 (77.1)	0.356
Simultaneous HIV diagnosis	10/28 (35.7)	30/57 (52.6)	0.142
Months since HIV diagnosis^a^; median (IQR)	18.1 (5.6–70.7)	28.1 (5.1–102.7)	0.622^b^
Previous OI and HIV-treatment			
Previous tuberculosis	4/28 (14.2)	4/57 (7)	0.281
Previous disseminated MAC	1/28 (3.5)	1/57 (1.7)	0.603
Previous disseminated histoplasmosis	1/28 (3.5)	3/57 (5.2)	0.729
CD4 count (cells/mm^3^); median (IQR)	71 (24–120.5)	19 (7–34)	<0.001^b^
Plasma HIV RNA (copies/mm^3^); median (IQR)	101,821 (2266.5–731,393)	159,000 (61,600–409,000)	0.199^b^
ART naïve	13/28 (46.4)	37/57 (64.9)	0.104
2 NRTI +1 NNRTI	8/15 (53.3)	13/20 (65)	0.486
2 NRTI + 1PI/r	3/15 (20)	5/20 (25)	0.527^c^
Other ART	4/15 (26.6)	2/20 (10)	0.367^c^
Empirical antifungal ≥ 72 hrs. before samples	7/28 (25)	13/57 (22.8)	0.823
Initial admission to critical care	2/27 (7.4)	2/56 (3.5)	0.445
Symptoms			
Fever	27/28 (96.4)	54/57 (94.7)	0.729
Weight loss	28/28 (100)	52/57 (91.2)	0.106
Diarrhea	17/28 (60.7)	30/57 (52.6)	0.481
Peripheral lymphadenopathy	14/28 (50)	27/57 (47.3)	0.819
Hepatomegaly	20/28 (71.4)	35/57 (61.4)	0.363
Splenomegaly	14/28 (50)	26/57 (45.6)	0.703
Mucosal lesion	3/28 (10.7)	7/57 (12.2)	0.833
Skin or soft tissue lesion	9/28 (32.1)	19/57 (33.3)	0.913
Laboratory abnormalities			
Anemia	20/28 (71.4)	41/57 (71.9)	0.962
Leukopenia	18/28 (64.2)	32/57 (56.1)	0.473
Thrombocytopenia	14/28 (50)	32/57 (56.1)	0.593
Total bilirubin (mg/dL), median (IQR)	0.8 (0.5–1.5)	0.7 (0.4–1.8)	0.643^b^
Alanine transaminase (UI/L), median (IQR)	33 (19–47)	46.5 (29–81)	0.024^b^
Aspartate transaminase UI/L), median (IQR)	67.5 (50–129)	152.5 (70–318)	0.008^b^
Lactic dehydrogenase (UI/L), median (IQR)	706 (325–1,249)	1,465 (742–2,894)	0.001^b^
Ferritin (ng/mL), median (IQR)	1,837.1 (976.7–2,060)	5,407.6 (1,759–15,000)	0.368^b^
Radiographic Pulmonary Involvement	18/28 (64.2)	45/57 (78.9)	0.147
Other opportunistic infection/neoplasia diagnosed	6/28 (21.4)	3/57 (5.2)	0.023^c^
Disseminated MAC	2/28 (7.1)	2/57 (3.5)	–
Disseminated *Cryptococcus neoformans* infection	1/28 (3.5)	0/57 (0)	–
Kaposi Sarcoma	1/28 (3.5)	1/57 (1.7)	–
Lymphoma	1/28 (3.5)	0/57 (0)	–
Disseminated Citomegalovirus	1/28 (3.5)	0/57 (0)	–
Extraintestinal *Salmonella* spp	1/28 (3.5)	0/57 (0)	–

Abbreviation: HUAg, histoplasma urine antigen; IQR, Interquartile Range; MAC, *Mycobacterium avium* complex; ART, Antiretroviral therapy; NRTI, Nucleoside Reverse Transcriptase Inhibitor; NNRTI, Non-Nucleoside Reverse Transcriptase Inhibitor; PI/r, Protease Inhibitor/ritonavir. If not otherwise indicated X^2^ test was applied.

^a)^ Excluding simultaneous HIV/disseminated histoplasmosis diagnosis;

^b)^ Mann-Whitney U-test;

^c)^ Fisher´s exact test.

### Clinical characteristics of cases with progressive disseminated histoplasmosis– negative patients with Histoplasma urinary antigen false positive results

The HUAg was positive in 5/203 (2.4%) of the PDH–negative patients; one of them was diagnosed with HIV simultaneously, and four had ART at the time of enrollment. All patients had blood and bone marrow cultures available. Three of these cases had radiographic pulmonary involvement. In only one case, the antifungal treatment was started ≥ 72 before obtaining the samples. Three patients had an alternative diagnosis: one was diagnosed with disseminated tuberculosis and two with disseminated *Cryptococcus neoformans* infection. Two patients remained without a diagnosis, one received empirical antifungals and survived, and the other died hours after enrollment. (Table 5).

**Table 5:** Clinical characteristics of patients with discarded progressive disseminated histoplasmosis with a false positive Histoplasma urine antigen.

Age/gender/ Enrollment date	Time since HIV diagnosis/Relevant medical history	CD4+(cells/ml)/Plasma RNA HIV(copies/ml)	Antirretroviral treatment	Symptoms	Radiographic pulmonary involment	Empirical antifungal ≥72 hrs. before samples	Sites sampled for culture	Final diagnosis/treatment/evolution
39/Male/ Sept-2016	39 mo./No	266/20	FTC+TDF+EFV	Weight loss, fever, anemia, thrombocytopenia, leukopenia, AST>2x, LDH >2x.	No	Yes	Blood culture (negative); Bone marrow culture (negative)	None/Empirical amphotericin/survived
28/Male/ Dec-2016	Simultaneous/No	NA	None	Weight loss, fever, peripheral adenopathy, skin lesions, LDH >2x, AST > 2x	Yes	Yes	Blood culture (*Cryptococcus neoformans*); Bone marrow culture (*C*. *neoformans*)	Disseminated cryptococcosis/amphotericin and fluconazole/survived
43/Male/ Oct-2016	193 mo./Tuberculosis, Pneumocystis;	8/20,300	ABC + 3TC + AZT + ATV/r	Weight loss, fever, diarrhea, hepatomegaly, peripheral adenopathy, bicytopenia, LDH >2x.	Yes	No	Blood culture (negative)+ Bone Marrow culture (negative)	None/Died without a diagnosis
33/Male/ Aug-2017	80 mo/*P*. *jirovecci* suspected	5/212,801	TDF + AZT + LPV/r	Weight loss, cough, diarrhea, fever, peripheral adenopathy, hepatomegaly, splenomegaly, AST >2x, LDH >2x.	Yes (budding tree)	No	Blood culture (*Mycobacterium tuberculosis*)+ Bone marrow culture (*M*. *tuberculosis*)	Disseminated tuberculosis/antituberculosis drugs /survived
27/male/ Oct -2017	82 mo./No	3/130,474	LPV/r+FTC+TDF+EFV	Diarrhea, weight loss fever, digestive tract bleeding, bicytopenia	Yes (Cavities)	No	Blood culture (*C*. *neoformans*) + Bone marrow culture (*C*. *neoformans*)	Disseminated cryptococcosis/amphotericin and fluconazole/survived

Abbreviation: FTC, emtricitabine; TDF, tenofovir; EFV, efavirenz; AST, aspartate transaminase; LDH, lactic dehydrogenase; ABC, abacavir; 3TC, lamivudine; AZT, zidovudine; ATV/r, atazanavir/ritonavir; LPV/r lopinavir/ritonavir

## Discussion

The results from this prospective multicenter and independent study evaluating the performance of the ALPHA-HUAg test in an endemic region for *H*. *capsulatum*, show that the test has a very high specificity (97%), it is easy to perform on-site without the need of sophisticated laboratory equipment and therefore faster than other methods. However, it lacks overall sensitivity (67%) when compared to the centralized latter generations of HUAg. [5] However, likelihood ratios obtained from our data show that HIV patients with and PDH are 27 times more likely to present a positive ALPHA-HUAg.

A higher sensitivity (79.3%) but lower specificity (75.7%) for ALPHA-HUAg was reported in a retrospective study including a mixed immunosuppressed population and used as a standard of reference urine samples deemed as positive by MiraVista-HUAg test and healthy controls. [9] Another study in Brazil reported a sensitivity of 100% and a specificity of 92.9% for ALPHA-HUAg in 8/78 proven PDH cases among AIDS patients.[11] The sensitivity and specificity of the ALPHA-HUAg found in this study are higher than in a previous report (sensitivity 22.7% and specificity 30%) from a subset of patients with culture-proven or positive histopathology cases of histoplasmosis and healthy controls.[12, 13] We also found a higher sensitivity and specificity of ALPHA-HUAg than in a study performed by MiraVista (sensitivity 44% and specificity 84%) using 50 positive urine samples with their assay, 25 urines from healthy volunteers and 25 from patients with discarded histoplasmosis as a standard of reference.[12] Another study, using a more recent kit from IMMY (IMMY *H*. *capsulatum* GM EIA), commercially available but to our knowledge not FDA approved, used as standard of reference urines determined as positive by MiraVista-HUAg and reported a similar sensitivity and specificity (64.5% and 99.8%) to the present study and also reported an improvement of the sensitivity (80.7%) by lowering the diagnostic cut-off. [14, 15] We acknowledge that by using the IMMY *H*. *capsulatum* GM EIA we could have observed a higher sensitivity of the HUAg, but we chose to test ALPHA Histoplasma EIA since it is FDA-approved and therefore likely to be approved in our and other regions.

In comparison with studies using different HUAg kits for the diagnosis of PDH the sensitivity found in ours is lower. Recently a multicenter report showed a sensitivity of 92.4% in 38 AIDS patients with PDH using the latest generation HUAg from MiraVista and a specificity of 99% estimated from healthy subjects and non-fungal infected patient urine samples.[16] The Centers for Disease Control and Prevention in the US developed a urinary antigen assay which was validated in an AIDS population in Colombia encompassing 28/106 patients with culture-proven histoplasmosis and showed a sensitivity of 84% and a specificity of 94% using samples from healthy volunteers or diagnosed with other infectious diseases. [17]

Irrespective of the HUAg kit used, our results are difficult to compare with previous studied, mainly due to our study design. Aforementioned studies use healthy subject’s urine as control, does not specify why the disease was suspected and often mix immunosuppressed population and offer almost none clinical information. However, we believe that the low sensitivity of ALPHA-HUAg found in this study is largely explained by the kit itself, the distinctive study design, and the use of a different standard of reference to estimate the diagnostic accuracy. However, other factors may have contributed as it is the great diversity of the *H*. *capsulatum* strains in our region, thus the different performance of the test outside North America. [18] This possibility has been previously approached during an outbreak of histoplasmosis due to a Latin America clade A class 6 strain in a colony of fruit bats which tested negative to the MiraVista antigen but further detected experimentally in mice infected by the same strain. [19] The same authors previously confirmed the utility of the MiraVista antigen in patients from Panama infected by the same Latin America clade A class 6 strain. [20] However, the performance of the ALPHA-HAUg is less studied in this region; although the report from Brazil informed 100% sensitivity, no information was provided regarding the infecting strains. [11] Additionally, while comparing the clinical characteristics of patients with IMMY-HUAg false-negative results with those considered true positives, we found a higher CD4 count, lower hepatic transaminases, and lactic dehydrogenase; this may be related to lower fungal burden, and somehow less tissue invasion. On the contrary, IMMY-HUAg false-negative cases, presented more frequently with other OI´s or AIDS-related malignancy (6/28, four of them in culture-proven PDH) which may have interfered with the HUAg performance, although not previously reported.

In this study, we found a high prevalence of proven-PDH (29.5%), using our suspected case definition and an extensive clinical and laboratory assessment, this is reflected by high positive predictive values of our test in our study. This should be considered while interpreting the results, since the usefulness of the test may vary depending on the disease prevalence and the suspect index of the physician requesting the test.

We analyzed the individual components of our standard of reference, of note bone marrow, showed a higher sensitivity than the IMMY-HUAg, while blood cultures and histopathological examination were similar. Bone marrow cultures are a valuable sample for the diagnosis of PDH and other resembling diseases (disseminated tuberculosis or disseminated MAC), but unfortunately—although complications are rare, and pain classified as low to moderate—physicians and patients perceive it as a painful, invasive procedure. We may have observed a higher sensitivity because we included many patients with hematological abnormalities, the situation in which is considered more useful.

We consider among the strengths of this study, the fact that we focused on a single immunosuppressed population since most of the studies mix transplant recipients, and immunocompetent patients. Additionally, our cases were mostly PDH since the majority resulted positive from blood and bone marrow cultures, while previous studies included more localized disease as pulmonary affection in which cases the sensitivity of the HUAg diminishes.[16] Additionally, we departed from a clinical definition of suspected PDH cases that reflects the daily clinical reasoning that triggers the diagnostic approach and allowed us to evaluate ALPHA-HUAg in a real-life scenario. Finally, we believe the high specificity of ALPHA-HUAg observed in this study is the most valuable information, since unlike many previous evaluations, were calculated specificity from PDH-negative instead of healthy controls. This challenged the ALPHA-HUAg to differentiate PDH from other resembling OI and even co-infections; these clinical scenarios are very frequent and challenging when treating HIV patients. In this regard, we consider that majority of five patients with discarded PDH presenting a positive HUAg were indeed false-positives because we found an alternative diagnosis in three of them and directed treatment lead to the clinical resolution. Of note, two of these cases were diagnosed with culture-proven disseminated cryptococcosis. Although cross-reactions with other fungal infections have been reported for the HUAg, *Cryptococcus neoformans* infection is not reported among them. [21] This warrants further study since both OIs are frequent among the HIV patients and targeted antifungal consolidation treatment is needed to avoid treatment failures. [22]

During the diagnostic approach of this study, we found 66 patients with other OIs and AIDS-defining malignancy, in nine instances co-infecting or co-existing patients with proven-PDH. Therefore, it should be advised that although non-culture technics as HUAg are non-invasive and provide faster results, in the HIV population, the culture-based approach and the tissue sampling for the histopathologic examination should not be replaced.

Our study has some limitations: despite an aggressive diagnostic workup, we may have missed some cases due to the biology of the disease and the lack of sensitivity of current fungal cultures. [4] Additionally, we did not perform molecular probe testing as recommended for positive cultures in order to distinguish from *Sepedomium* spp and *Chrysosporium* spp, fungi with similar macroconidia to *H*. *capsulatum*. [23] However, these saprophytic fungi rarely cause disseminated infection in humans, and most of the reports are from respiratory samples or considered as contaminants. [24, 25] Finally, although suggested as part of the diagnostic approach, we did not perform Histoplasma antibody testing, since it was not readily available, and we expected a low sensitivity in the HIV population. [23] Additionally, we failed to assess the history of food causing coloration of the urine, caffeine intake and the consumption of acetaminophen and acetylsalicylic acid; this is relevant since the manufacturer cites these substances as eliciting an “unknown” effect on the performance of the test. Finally, we did not assess the turnaround time for results, since the ALPHA-HUAg testing was performed in the central laboratory and processed in batches. However, our experience with the on-site use of the test was positive; it was easy to standardize, required only a short technician training and the turnaround result time for an entire batch was about four hours. Thus, we believe it can be performed in most of the second and third level hospitals in Latin America.

In conclusion, ALPHA-HUAg is a useful test for the diagnosis of PDH—in patients with high level of suspicion in endemic regions for *H*. *capsulatum*–and may favor early targeted-antifungal treatment, thus improving the prognosis of these frail HIV-patients. Nevertheless, the culture-based approach should ensue simultaneously since co-infections are frequent and a negative result of ALPHA-HUAg does not discard PDH in patients from endemic regions for *H*. *capsulatum*.

## Supporting information

S1 Checklist STARD checklist. (DOCX)
